# The impacts of COVID-19 hospitalizations on non-COVID-19 deaths and hospitalizations: A panel data analysis using Brazilian municipalities

**DOI:** 10.1371/journal.pone.0295572

**Published:** 2023-12-14

**Authors:** Naercio Menezes-Filho, Bruno Kawaoka Komatsu, Luana Villares

**Affiliations:** 1 Ruth Cardoso Chair, Insper, São Paulo, São Paulo, Brazil; 2 School of Economics, Business, and Accounting, University of São Paulo, São Paulo, São Paulo, Brazil; Universidade de Sao Paulo, BRAZIL

## Abstract

The COVID-19 pandemic in Brazil has brought many challenges, particularly regarding the management of hospital capacity, and a new demand for healthcare that added to the preexisting demands, such as neoplasms, cardiovascular diseases and births. In this paper, we estimate the impact of the pandemic on the number of deaths and hospitalizations for other diseases. We construct a monthly panel data of deaths and hospitalizations for various causes by the municipality of residence and relate them to COVID-19 hospitalizations using regression models that control for municipalities fixed-effects and interactions between State and month fixed-effects. The standard errors are clustered at the municipality level. Our estimates imply that 100 more hospitalizations by COVID-19 is associated with a drop of 49 non-COVID-19 hospitalizations and an additional four deaths for other reasons (all measured per 100,000 pop.). The impact of intensive care units COVID-19 hospitalizations on mortality is larger. The groups most affected are the African Brazilians, less-educated and the elderly. Additional deaths occurred both at households and at hospitals. The main causes of additional deaths were diseases related to the circulatory and endocrine system. The decline in hospitalizations for other causes seems to be related to the overcrowding of hospitals in periods of surge in the COVID-19, alongside with the fall in the demand for care by the citizens who were afraid of COVID-19 infection. These mechanisms affected more strongly the vulnerable groups of the population. Our results highlight the importance of promoting the awareness of heightened risk of non-communicable chronic diseases during a health emergency context. This should be done preferably through already established channels with community outreach, such as the Family Health Program in Brazil.

## 1 Introduction

The Coronavirus disease 2019 (COVID-19) pandemic has brought additional challenges to health systems worldwide, by overcrowding hospitals and increasing the demand for specialized health professionals, drugs and other limited resources [1, 2]. Difficulties were more severe in low- and middle-income countries, which often have overcrowded health systems [3]. In addition, some low- and medium-income countries, such as Brazil, have relatively larger shares of the population in situations of social vulnerability and living in informal urban settlements, which have restrictions on sanitation and physical space [4].

Brazil has the one of the largest public health systems in the world, the Brazilian Unified Health System (SUS). Despite being underfunded, its primary care teams cover 165 million people (77% of the population) and almost all municipalities in the country in 2023 [5]. The primary care level operates based on 55 thousand multidisciplinary family health teams, whose actions are centered on visit of registered families and intersectoral initiatives of health promotion and disease prevention [5]. One of the main characteristics of SUS is universal access, including people from other nationalities, free of charge, and the provision of primary, secondary, and tertiary care to all citizens as a constitutional right [6, 7]. This way, SUS provides access to families that otherwise might not be able to pay for healthcare in private facilities. However, persons with higher incomes and access to health insurance tend to access more private facilities to avoid the challenges of the public system, which includes waiting lines for consultations and exams.

With systems characterized by universal access to care, such as SUS, the difficulties of increased demand on the health system might have been exacerbated as a result of the COVID-19 pandemic. As any person is allowed to attend public health care facilities, persons exhibiting symptoms or infected persons would make use of facilities might use healthcare facilities, whereas people with chronic or treatable conditions might refrain from going to health facilities out of fear of becoming infected.

Brazil was the second country with the highest number of confirmed deaths by COVID-19 until the end of 2022, when it reached 619,334 deaths [8]. Waiting lines in the Brazilian public healthcare system were already long before COVID-19, and the pandemic has aggravated this problem [9]. In the beginning of the pandemic period, hospitalizations in intensive care units (ICU) in Brazil surged from approximately 69,000 in February 2020 to 88,000 in August, and in some State capitals occupation rates reached 100% in 2020 [10, 11].

Previous research has calculated the all-cause excess mortality during the COVID-19 pandemic period, as a measure of the total burden of the pandemic that overcomes measurement errors due to insufficient testing [12]. Brazil appears in the 5^th^ position of excess deaths (681 thousand) among the 194 WHO members between 2020 and 2021, which represents 24% of the expected number of deaths for the period [12]. Besides the deaths directly caused by COVID-19 infection, however, the pandemic may also indirect affect mortality by other causes, by reducing the access to hospital care for several reasons [13, 14]. On the supply side, the pandemic reduced medical resources and hospital beds for non-COVID-19 patients, and increased delays in treatment and surgeries [15, 16]. Moreover, in places where the health system was at the risk of collapsing, social isolation measures against the COVID-19 spread and hospital suspended elective surgeries during peaks of the pandemic, which increased barriers for non-COVID-19 patients to access healthcare [17].

On the demand side, sick people may have adjusted their behavior, avoiding healthcare facilities out of fear of being contaminated [17, 18]. These mechanisms are likely to have increased delays in the treatment of diseases, so that complications and acute continuation of those conditions may have led to an increase in mortality [18].

For example, in the case of the city of Manaus (capital of Amazonas, in the north of Brazil), during 2020 and 2021, the COVID-19 ICU hospitalization rate peaked twice, in May and June 2020 (reaching approximately 16 hospitalizations per 100 thousand population) and around January 2021 (reaching 30 hospitalizations per 100 thousand population). During those peaks, non-COVID-19 ICU hospitalization rate dropped from its average level of around 23 hospitalizations per 100 thousand population to, respectively, 13 and 15 hospitalizations per 100 thousand population. Non-COVID-19 mortality rate also deviate from its average (around 40 deaths per 100 thousand population) during those peaks: in May 2020 and January 2021, it increased, respectively, to 67 and 57 deaths per 100 thousand population. These patterns inform the rationale of the study and highlight that when ICU hospitalizations by COVID-19 increased, non-COVID-19 ICU hospitalizations decreased and, therefore, non-COVID-19 mortality also increased.

As such, the aim of the study was to directly estimate the impacts of COVID-19 hospitalizations on non-COVID-19 mortality and hospitalizations in Brazilian cities during the first year of the pandemic. We use a longitudinal ecological design, perform heterogeneity analysis, and evaluate the channels that transmit the impact from COVID-19 hospitalizations to non-COVID-19 mortality and hospitalizations with a monthly panel of municipalities to estimate the impact of the rate of COVID-19 hospitalizations of people living in a municipality on the rates of non-COVID-19 mortality and hospitalizations among its population, controlling for the municipality-specific heterogeneity that is invariant over time and for State-level aggregate factors that may vary over time.

## 2 Materials and methods

### 2.1 Study design

This paper uses a longitudinal ecological design with a monthly panel of Brazilian municipalities to investigate the impact of the rate of COVID-19 hospitalizations on the rates of non-COVID-19 hospitalizations and deaths. Our panel includes 5,569 municipalities over 12 months, adding to 66,828 observations. Our analysis calculates hospitalizations and deaths based on the municipality where the patients were residing (in the case of hospitalizations data) or where the deceased lived (in the case of mortality data), so that we correctly capture the geographic relationship between COVID-19 spread and non-COVID-19 deaths and hospitalizations.

### 2.2 Variables

The primary variables analyzed in our study are the non-COVID-19 mortality and hospitalization rates and the COVID-19 hospitalization rate, all expressed as rates per 100,000 population, for each municipality of residence and by month. In Brazil, the data on number of hospitalizations and deaths depend on the registry of the government information system, which has good quality in general, even when compared to mortality data collected in the 2010 Demographic Census [19]. To compute the non-COVID-19 rates, we exclude deaths and hospitalizations caused by respiratory diseases, pregnancy-related causes, and unspecified causes. We exclude respiratory diseases because they may include misclassified COVID-19 cases, especially in the early stages of the pandemic [20]. Supporting evidence for this is presented in S1 Text.

In further analysis, we calculate non-COVID-19 mortality rates by disease groups, place of death, and socioeconomic characteristics of the deceased, using the ratio of the number of deaths in a particular group in a given municipality and month to the total population of that municipality in 2020. Not all observations in our sample have information on place of death, resulting in a reduced sample size of 64,185 observations with valid data for all-cause mortality by place of death and 63,200 observations with valid data for non-COVID-19-related deaths by place of death.

We categorize deaths and hospitalizations into disease groups based on their primary causes, as defined by the 10th edition of the International Statistical Classification of Diseases and Related Health Problems. COVID-19 cases were identified by code B34.2.

### 2.3 Data sources

We obtain hospitalization data from two major sources. The Hospital Information System provides data on hospitalizations funded by the Brazilian public health system, the Unified Health System (SUS) [21]. SUS provides access to health services in public health facilities, but it also funds services in private facilities, all of which are included in the database. The remaining hospitalizations are paid for by private health insurance companies, and we obtained this information from the National Agency of Supplementary Health Care [22]. We use variables from both datasets that store information on the municipalities of residence and admission, date of admission, main diagnose (coded with the 10^th^ revision of the International Statistical Classification of Diseases and Related Health Problems–ICD-10), and whether the patient was taken to ICU

The data on deaths were gathered from the Brazilian Mortality Information System, which covers all occurrences in Brazil that were registered through death certificates [23]. This database includes information on the date, cause and place of death, socioeconomic information of the deceased, which we use to identify subgroups, and the municipality of residence of the deceased and of the occurrence. The population data are estimations of the population by municipality as of July 1st, 2020, carried out by the Brazilian Institute of Geography and Statistics. They exclude COVID-19 deaths in 2020, as they are based on data from the Demographic Censuses and international migration [24].

### 2.4 Statistical analysis

We examine whether COVID-19 hospitalizations have led to a decrease in non-COVID-19 hospitalizations and to increased non-COVID-19 mortality. This could be due to a reduction in the supply of healthcare resulting from hospital overload, limited hospital resources and capacity, as well as government social isolation measures. Patient’s behavior, such as avoiding hospitals due to fear of COVID-19 infection, could have led to missed opportunities for preventive care or delayed treatment of non-urgent conditions, potentially worsening health outcomes and contributing to increase in deaths.

Our statistical analysis employs a panel data fixed-effects model to estimate the impact of COVID-19 hospitalization rates on non-COVID-19 mortality and hospitalization rates. The main challenge is to isolate the impacts of COVID-19 hospitalizations from other factors that may have affected both the COVID-19 hospitalizations and the outcome variables. For example, municipalities with greater proportions of senior people or poor living conditions and sanitation infrastructure might experience higher rates of COVID-19 hospitalizations and also deaths from other causes. These characteristics do not vary quickly over time, so that we address this issue by exploring the panel structure of our data and control for the effects of specific municipality characteristics that are invariant over time and time-varying State effects. For each municipality m, in State s and month t, the estimated equation is:

$$Y_{mst}=\beta COVIDHosp_{mst}+\alpha_{m}+\delta_{st}+\varepsilon_{mst}$$ (1)

where *Y*_*mst*_ is the outcome (non-COVID-19 rates of hospitalizations or mortality), *COVIDHosp*_*mst*_ is the rate of hospitalizations by COVID-19, and *β* is our parameter of interest. *α*_*m*_ is a vector of municipality fixed effects. *δ*_*st*_ is a vector of State-month fixed effects that control for State-level characteristics that vary over time, such as State non-pharmacological measures against the pandemic and economic shocks, for example. Finally, *ε*_*mst*_ is a random error term. All our regressions are weighted by the population and are performed using the the statistical program R’s version 0.11.1 of the package fixest [25]. We report 95% confidence intervals.

We conduct additional analyses to provide more insight into our main results. The analysis included: 1) whether urgent cases have different impacts compared to the overall cases, using the rate of COVID-19 hospitalizations in ICU as our primary explanatory variable; 2) how non-COVID-19 mortality rates in hospitals and households responded to increases in COVID-19 hospitalizations; 3) whether COVID-19 hospitalizations displaced individuals in critical conditions to seeking hospital care in other municipalities. To examine which socioeconomic groups were most indirectly affected by COVID-19, we also carry out a heterogeneity analysis of impacts according to age groups, race, and schooling level. Last, we explore the causes of non-COVID-19 mortality that were more responsive to increases in COVID-19 hospitalizations.

## 3 Results

Table 1 presents descriptive statistics of the main variables used in our analysis and reports the mean and standard deviation of our database of municipalities over time. A few stylized facts emerged. First, the average number of hospitalizations by non-COVID-19 causes were greater than those by COVID-19. There were on average 19.2 hospitalizations for other causes for each one COVID-19 hospitalization. Moreover, COVID-19 demanded on average relatively more ICU beds than other causes: ICU hospitalizations represented 24.5% of the total COVID-19 hospitalizations, but only 7% of non-COVID-19 hospitalizations. The ratio of average deaths per hospitalization was greater for COVID-19 (0.34) than for other causes (0.10).

**Table 1:** Descriptive statistics of rates of mortality and hospitalizations (n = 66,828). Brazil, 2020*.

Variable	Mean	Std. Dev.
COVID-19 Hospitalizations	24.9	30.2
Non-COVID-19 Hospitalizations	476.9	157.5
COVID-19 ICU Hospitalizations	6.1	8.5
Non-COVID-19 ICU Hospitalizations	33.8	20.5
COVID-19 Deaths	8.4	11.7
Non-COVID-19 Deaths	47.2	15.5
Population (1,000 pop.)	37.5	219.2

* per 100,000 population

We report hospitalizations and mortality rates disaggregated by socioeconomic groups in Table 2. Separating rates by age groups allows us to verify that the ratio of non-COVID-19 to COVID-19 hospitalizations was greater among the younger groups (80 and 106, respectively, among children and the youth) in contrast with older groups (19 and 9, respectively, among adults and seniors). The proportion of COVID-19 hospitalizations that end up in ICU on average (17% among children, 18% among the youth, 21% among adults, and 28% among seniors) and the ratio of average deaths per hospitalization (0% among children, 9% among the youth, 18% among adults, and 48% among seniors) were higher, the older the group. However, the proportion of non-COVID-19 ICU hospitalizations among all non-COVID-19 hospitalizations were higher among the children and the seniors (13% among children, 2% among the youth, 5% among adults, and 12% among seniors).

**Table 2:** Descriptive statistics of rates of mortality and hospitalizations by socioeconomic groups. Brazil, 2020*.

Group	Variable					
	Covid-19 Hosp.	Non-COVID-19 Hosp.	COVID-19 ICU Hosp.	Non-COVID-19 ICU Hosp.	COVID-19 Deaths	Non-COVID-19 Deaths
**Age Groups**						
Children (0 yrs to 14 yrs)	0.6	48	0.1	6.2	0	1.6
Youth (15 yrs to 29 yrs)	1.1	116.3	0.2	2.6	0.1	2.5
Adults (30 yrs to 59 yrs)	10.2	190.1	2.1	9.9	1.8	11.4
Seniors (60 yrs or more)	13	122.5	3.7	15.1	6.3	31.4
Not Available	0	0	0	0	0	0
**Education groups**						
Less than Middle School	-	-	-	-	3.3	21.9
Middle School	-	-	-	-	1.2	7.1
High School	-	-	-	-	1.6	6.7
College	-	-	-	-	0.6	2.2
Not Available	-	-	-	-	1.5	9.1
**Race groups**						
African Brazilian or Indigenous People	-	-	-	-	3.9	22.7
Caucasian or Asian People	-	-	-	-	4.1	23.2
Not Available	-	-	-	-	0.3	1.2

* per 100,000 population

Data of hospitalizations financed privately do not have information on race and education, so we were not able to proceed to the same comparisons. However, we observe that the ratio of non-COVID-19 deaths to the COVID-19 deaths was higher among the less educated (6,6 among those with less than Middle school and 3,7 among those with a college degree). Disaggregation by race groups lead to similar results among groups.

Table 3 reports the main results of this paper, showing that increases in the rate of COVID-19 hospitalizations in a month among people living in a municipality were associated with declines in the rate of non-COVID-19 hospitalizations and with rises in non-COVID-19 mortality rates among its population. Estimates of columns (1) and (2) do not control for State-month interactions, while columns (3) and (4) do adjust for these effects. Estimates imply that 100 more hospitalizations by COVID-19 (per 100,000 pop.) provoked a fall of 51 non-COVID-19 hospitalizations and an increase of four deaths for other reasons (per 100 thousand pop.). The differences in magnitudes when controlling for State-month fixed effects indicate that there are relevant time-varying State-level factors that were confounding the relationship between hospitalizations by COVID-19 and our outcomes.

**Table 3:** Impacts of hospitalizations by COVID-19 on mortality and hospitalizations by other causes (n = 66,828). Brasil, 2020.

	Unadjusted		Adjusted	
	Hospitalizations	Mortality	Hospitalizations	Mortality
	(1)	(2)	(3)	(4)
Hospitalizations	-1.21	0.048	-0.510	0.043
by COVID-19	[-1.45; -0.975]	[0.036; 0.061]	[-0.598; -0.423]	[0.033; 0.052]
R^2^	0.735	0.429	0.874	0.456
Within R^2^	0.143	0.012	0.029	0.005
Elasticity	-0.063	0.026	-0.027	0.023

Table 4 shows results using the rate of ICU hospitalizations by COVID-19 as the explanatory variable. An increase of 100 ICU hospitalizations by COVID-19 was associated with a reduction of 7 non-COVID-19 ICU hospitalizations (column 1) and an increase of 9 non-COVID-19 deaths (column 2). The impacts on mortality of ICU hospitalizations were larger than that of the overall COVID-19 hospitalizations (in column 4 of Table 3), which reflects the fact that ICU hospitalizations were urgent cases that had a higher probability of death. But, the result of column (1) also confirms that the impact of COVID-19 on ICU hospitalizations for other reasons was smaller than that on the overall hospitalizations, as it was more difficult to postpone non-COVID-19 hospitalizations when conditions were more serious.

**Table 4:** Impacts of COVID-19 ICU hospitalizations on non-COVID-19 ICU hospitalizations and mortality (n = 66,828). Brasil, 2020.

	Dependent Variable	
	ICU Hosp.	Mortality
	(1)	(2)
ICU Hospitalizations	-0.070	0.092
by COVID-19	[-0.094; -0.047]	[0.069; 0.116]
R^2^	0.758	0.454
Within R^2^	0.002	0.002
Elasticity	-0.013	0.012

We further investigate how COVID-19 hospitalizations impacted non-COVID-19 hospitalizations and mortality. First, Table 5 shows that COVID-19 hospitalizations affected more the deaths in households then in hospitals. It reports that an increase of 100 hospitalizations by COVID-19 (per 100,000 pop.) caused 2.4 additional non-COVID-19 deaths in households and 1.5 deaths in hospitals per 100 thousand population. Additionally, we investigate how individuals with severe medical needs have been displaced by COVID-19-related. Table 6 shows the impact of the rate of ICU hospitalizations by COVID-19 on the share of non-COVID-19 deaths away from the municipality of residence by municipality size. The first row shows that in the overall sample, for each 100 additional COVID-19 ICU hospitalizations, the percentage of non-COVID-19 deaths away from the municipality of residence increased, albeit not significantly, 1.7 percentage points (pp.) and the percentage of all-cause deaths, including COVID-19 cases, increases 10 pp. The result on non-COVID-19 mortality was significant only at the 10% level. The other rows of the overall sample results indicate that the result above was completely explained by the deaths at hospitals, as the estimates for deaths at households were small and not statistically significant. These results suggest that increased demand for ICU beds due to COVID-19 moved not only COVID-19 patients, but also other patients with severe conditions towards hospitals in other municipalities.

**Table 5:** Hospitalizations by COVID-19 and non-COVID-19 (per 100 thousand population) by place of death (n = 66,828). Brasil, 2020.

	Dependent Variable:	
	Deaths per 100,000 Pop.	
	Households	Hospitals
	(1)	(2)
Hospitalizations	0.024	0.015
by COVID-19	[0.020; 0.028]	[0.009; 0.021]
R^2^	0.418	0.507
Within R^2^	0.005	0.001
Elasticity	0.051	0.012

**Table 6:** Impacts of ICU hospitalizations by COVID-19 on the percentage of deaths away from the municipality of residence. Brasil, 2020.

	Non-COVID-19 Mortality Rate (n = 63,200)	All-Cause Mortality Rate (n = 64,185)
All	0.017 [-0.0007; 0.034]	0.104 [0.087; 0.122]
Households	0.001 [-0.001; 0.004]	-0.0006 [-0.002; 0.001]
Hospitals	0.016 [-0.0003; 0.033]	0.115 [0.097; 0.133]

We examine the relative impacts of COVID-19 hospitalizations on non-COVID-19 mortality of different socioeconomic groups in Table 7. Estimates separated by levels of education indicate that the impact on mortality was the largest among those with less than middle school. When we estimate separately by racial groups, estimates imply that an increase of 100 additional COVID-19 hospitalizations was associated with a rise of 3 deaths due to non-COVID-19 among African Brazilians or Indigenous people, and 2 deaths among Caucasian or Asian people. We also show the heterogeneity of effects by age group in Table 7. Older age groups had a relatively larger impact of COVID-19 on non-COVID-19 mortality. We observe that while the impact was not statistically significant among children, it grew with age group and reached an additional 3.6 deaths among seniors.

**Table 7:** Impacts of hospitalizations by COVID-19 on non-COVID-19 deaths by socioeconomic characteristics (n = 66,828). Brasil, 2020.

	Estimate
Less than Middle School	0.023 [0.018; 0.028]
Middle School	0.004 [0.002; 0.007]
High School	0.006 [0.003; 0.008]
College	0.001 [0.0004; 0.002]
African Brazilians or Indigenous people	0.026 [0.020; 0.032]
Caucasian or Asian people	0.015 [0.010; 0.021]
Children (0 yrs to 14 yrs)	0.0005 [-0.0004; 0.001]
Youth (15 yrs to 29 yrs)	0.002 [0.0009; 0.003]
Adults (30 yrs to 59 yrs)	0.005[0.002; 0.007]
Seniors (60 yrs or more)	0.036 [0.028; 0.043]
Total	0.043 [0.033; 0.052]

We investigate which groups of diseases were most affected by the COVID-19 hospitalizations. Table 8 shows that the groups of diseases most impacted in terms of hospitalizations were diseases of the digestive system, circulatory system, and genitourinary system. The estimates imply that 100 additional COVID-19 hospitalizations led to declines of 9, 7, and 7 hospitalizations per 100 thousand population, respectively.

**Table 8:** COVID-19 hospitalizations and hospitalizations and mortality by specific causes (per 100 thousand population) (n = 66, 828). Brasil, 2020.

	Hospitalizations	Mortality
Circulatory	-0.068 [-0.079; -0.058]	0.016 [0.011; 0.020]
Endocrine	-0.009 [-0.013; -0.005]	0.009 [0.007; 0.011]
Nervous	-0.013 [-0.017; -0.009]	0.003 [0.002; 0.004]
External	-0.042 [-0.051; -0.033]	0.002 [0.0003; 0.004]
Infectious	-0.024 [-0.037; -0.011]	0.002 [0.0003; 0.003]
Neoplasms	-0.041 [-0.050; -0.032]	0.002 [0.0002; 0.004]
Genitourinary	-0.066 [-0.078; -0.053]	0.0008 [-0.0001; 0.002]
Digestive	-0.088 [-0.101; -0.074]	0.0002 [-0.0009; 0.001]
Musculoskeletal	-0.025 [-0.030; -0.019]	4.89e-5 [-0.0003; 0.0004]

As for the impact of COVID-19 hospitalizations on deaths, Table 8 shows that diseases of the circulatory system, endocrine, nutritional, and metabolic diseases, and diseases of the nervous system were the groups most impacted. An increase of 100 hospitalizations by COVID-19 (per 100,000 pop.) increased the mortality in those groups by 2, 1 and 0.3, respectively. Hospitalizations of digestive, genitourinary and musculoskeletal diseases were negatively affected by COVID-19 hospitalizations, but this impact did not translate into an increase in mortality by those same groups.

## 4 Discussion

Our results indicate the rate of hospitalizations by COVID-19 affected non-COVID mortality and hospitalizations in the Brazilian municipalities during 2020. An increment of 100 COVID-19 hospitalizations was associated with a decrease of 50 non-COVID-19 hospitalizations, and increase of 4 non-COVID-19 deaths, all measured per 100,000 population.

To deepen our comprehension of our primary findings, we conducted supplementary analysis. First, we confirm that non-COVID-19 mortality increased more in households than in hospitals during times of high COVID-19 hospitalizations. This may be due to hospital overcrowding, which could prevent urgent patients from receiving medical care, as well as preventable or treatable diseases escalating into fatal conditions at home. Second, there was little evidence that COVID-19 ICU hospitalizations increased the percentage of non-COVID-19 deaths away from the individual’s municipality of residence. This result might indicate that COVID-19 increased the displacement of people needing medical care from their home municipalities, potentially due to scarcity of hospitals resources related to the increased demand from COVID-19 cases. Another possible explanation is that the centralized organization of hospitals in Brazil might make people with COVID-19 symptoms go to other municipalities to seek ICU healthcare.

The heterogeneity of the effects shows that the groups that were more affected by COVID-19 hospitalizations in terms of mortality were people with lower schooling levels, African Brazilians or Indigenous people, and older people. Moreover, the disease groups that drove the overall hospitalization results were diseases of the digestive, circulatory, and of the genitourinary systems. As for the mortality results, metabolic diseases, diseases of the circulatory system, endocrine, nutritional, and diseases of the nervous system were the most affected. Previous research found that older age and the incidence of heart diseases and diabetes mellitus are risk factors for COVID-19, so that it is possible that an additional effect of fear among people with those characteristics also affected their non-COVID-19 mortality [26].

One limitation to our study is that our data do not allow us to disentangle how much of the impact is due to scarcity of resources because of the overcrowding of hospitals and how much is due to the individuals adjusting their behavior due to COVID-19. Also, our data do not allow us to distinguish between the impacts of COVID-19 hospitalizations on the short-term mortality of patients with more serious conditions and the longer-term mortality related to the worsening of preventable or treatable cases due to lack of access to healthcare. Moreover, non-COVID-19 mortality might still be directly related to COVID-19 infection, as COVID-19 might increase mortality by other conditions. In this case, the rise of COVID-19 hospitalizations would also affect mortality directly. Future research may focus on these points that remain unsolved.

This study provides evidence of the COVID-19’s impact on the non-COVID-19 hospitalizations and mortality, using a direct estimation approach. Our findings show a clear dose-response relationship between COVID-19 hospitalizations and non-COVID-19 hospitalizations and mortality across municipalities and time. These results complement the existing literature and enhance our understanding of how excess mortality during the pandemic is not entirely explained by deaths by COVID-19 infection. Prior studies examine changes in non-COVID-19 hospitalizations and mortality from specific causes during the pandemic, comparing them to pre-pandemic periods. Such studies have shown a decline in non-COVID-19 hospitalizations and an increase in mortality during the pandemic using data from high-income countries and from low- and medium-income countries. Two meta-analyses find significant increase in non-COVID-19 excess mortality [13, 14]. One of them compares all-cause excess mortality to non-COVID-19 excess mortality and concludes that an important share of the total excess mortality is due to indirect non-COVID-19 mortality [14]. The other meta-analysis indicates that declines in acute care hospitalizations, increases in morbidity and in disruptions in health care [13]. This literature is still expanding and more recent studies reach similar conclusions [27–31]. Our results confirm that COVID-19 had an indirect significant impact on non-COVID-19 mortality and hospitalizations.

Our results also reinforce previous evidence on the impacts of COVID-19 on mortality and health services. During the COVID-19 pandemic, detection and treatment of other diseases declined, contributing to an increase in out-of-hospital mortality [15, 31, 32]. Moreover, it may also have contributed to increased proportions of more serious conditions, which associated with increases delays in hospital procedures might help explain in-hospital mortality [15, 16]. We find that cardiovascular and endocrine diseases are two of the groups most impacted by the pandemic, as in a previous study [30]. Those are also two of the chronic non-communicable diseases with the highest prevalence in the Brazilian population and people suffering from those conditions are highly vulnerable to changes in access to healthcare [33].

We find that the groups most indirectly affected by the pandemic were people with lower schooling levels, black or Indigenous people, and older people, which were the same as in previous studies that estimated the COVID-19 excess mortality [14]. Studies indicate that low-socioeconomic groups are disproportionately susceptible to COVID-19, and the pandemic exacerbated the difficulties in their accessibility to health services [14, 34]. In Brazil, there is a high correlation between race and poverty, and the latter is associated with more vulnerability [35].

The results in this article shed light on possible paths for public policy. We highlight the importance of promoting the awareness of the increased risk of other diseases during a pandemic, especially the non-communicable chronic diseases, whose treatment cannot be suspended. Information should be disseminated to families covered by the Family Health Strategy, a large primary care program with home visits in Brazil [36], and medical appointments may benefit from expansion in the adoption of telemedicine [37]. Moreover, we bring evidence of indirect impacts of the COVID-19 hospitalizations on postponement or cancelling elective treatments, which may lead to an increase in deaths and other health issues in the future.

## Supporting information

S1 Text Respiratory diseases. (DOCX)

S2 Text ICU hospitalizations. (DOCX)

S1 Table ICU hospitalizations by COVID-19 and ICU hospitalizations by respiratory and other causes (per 100 thousand population). (DOCX)
