# Supplementary material for: The impacts of COVID-19 hospitalizations on non-COVID-19 deaths and hospitalizations: A panel data analysis using Brazilian municipalities
Source: PLoS One. 2023 Dec 14;18(12):e0295572. doi: 10.1371/journal.pone.0295572 (PMC10721066; doi:10.1371/journal.pone.0295572)
Supplement: S1 Text — (DOCX) [file pone.0295572.s001.docx]

## S1. Appendix – Respiratory diseases

We regressed ICU hospitalizations for respiratory diseases on ICU hospitalizations for COVID-19 and obtained a positive coefficient, in contrast with the results obtained when doing the same estimation using the rate of hospitalizations for other diseases. Given the similarity of symptoms between COVID-19 and other respiratory disorders and the lack of COVID-19 tests in Brazil, it is possible that the positive estimate is due to misreporting of COVID-19 cases.
