# Supplementary material for: The impacts of COVID-19 hospitalizations on non-COVID-19 deaths and hospitalizations: A panel data analysis using Brazilian municipalities
Source: PLoS One. 2023 Dec 14;18(12):e0295572. doi: 10.1371/journal.pone.0295572 (PMC10721066; doi:10.1371/journal.pone.0295572)
Supplement: S2 Text — (DOCX) [file pone.0295572.s002.docx]

## S2. Appendix – ICU hospitalizations

ICU hospitalizations were also extracted from the databases of SIH-SUS and ANS. They were identified, respectively, by the variables ‘UTI MES TO’ and ‘QT DIARIA UTI’ that report the number of days the individual rested in ICU beds. We considered as ICU hospitalization the observations that assumed positive numbers in those variables, meaning that all cases that rested at least one day in an ICU were accounted among ICU cases, even though the individual may have rested other days in common beds.
