# Supplementary material for: The impacts of COVID-19 hospitalizations on non-COVID-19 deaths and hospitalizations: A panel data analysis using Brazilian municipalities
Source: PLoS One. 2023 Dec 14;18(12):e0295572. doi: 10.1371/journal.pone.0295572 (PMC10721066; doi:10.1371/journal.pone.0295572)
Supplement: S1 Table — (DOCX) [file pone.0295572.s003.docx]

**Table S1: ICU Hospitalizations by COVID-19 and ICU Hospitalizations by Respiratory and Other Causes (per 100 Thousand Population)**

|  | Dependent Variable: | |
| --- | --- | --- |
|  | ICU Hosp. per 100,000 Pop. | |
|  | Respiratory | Other |
|  | (1) | (2) |
| ICU Hospitalizations by COVID-19 | 0.028  [0.020; 0.036] | -0.070  [-0.094; -0.047] |
| Observations | 66,828 | 66,828 |
| R^2^ | 0.443 | 0.758 |
| Within R^2^ | 0.002 | 0.002 |
| Elasticity | 0.041 | -0.013 |

Data are estimates of the impact of COVID-19 ICU per 100,000 population cause-specific ICU hospitalizations per 100,000 population. 95% CI based on standard-errors clustered at the municipality level in square brackets. Data from 2020 based on municipality of residence.
